# La importancia de los relatos de vida en la atención a una buena muerte

**DOI:** 10.23938/ASSN.1134

**Published:** 2025-09-26

**Authors:** Geno Ochando Ortiz

**Affiliations:** Gobierno de Navarra Departamento de Salud Servicio de Humanización, Aseguramiento y Coordinación Interdepartamental Pamplona España

En este número de la revista Anales del Sistema Sanitario de Navarra se ha publicado la colaboración especial *Que te aproveche mirar lo que miras: Una reflexión humanista sobre la mirada paliativa*, de Juan Luis Torres-Tenor^1^. En ella, tras sus reflexiones, el autor concluye que encuentra consuelo al pensar que *siempre nos quedará la medicina centrada en la persona. La humanización. Una atención holística. Cuidados paliativos*^1^.

Desde el año 2018 Navarra ha sentado las bases de un modelo de atención que humanice la atención en salud. Tras la primera *Estrategia de humanización del sistema sanitario público de Navarra*, presentada en 2018 y atravesada por la pandemia de COVID-19, se ha impulsado la Estrategia 2024-2028^2^, fruto de un proceso de evaluación participativa donde profesionales, asociaciones de pacientes y ciudadanía en general han marcado las líneas de trabajo futuras. Quizás lo más destacable de esta evaluación es la contundencia con la que emergen la necesidad de la línea de cuidado y bienestar profesional, junto con la de participación, y el nacimiento y el final de vida como etapas de la vida a las que debíamos prestar especial atención.

La humanización en la etapa final de la vida implica prestar una especial atención, si cabe, a la escucha, por la situación de desprotección en la que nos encontramos, y que nos impide *negociar*, *pedir*, *consensuar* las opciones que profesionales sanitarios, que el propio sistema sanitario, nos ofrecen. Entran en juego la dignidad, la despersonalización unida al miedo, la vulnerabilidad … que hacen que siempre el paciente esté en una situación *de desventaja*. Existe, nos guste o no, un desequilibrio de poder, mediatizado por el miedo a la muerte, que dificulta la toma de decisiones por parte de la persona atendida a pesar de que, sobre el papel, desde 2010 diferentes leyes nacionales y autonómicas garantizan la autonomía del paciente^3^^-^^5^.

Y luego están las y los profesionales sanitarios, como actores de la misma escena. El autor de la colaboración especial hace referencia a la *obligación del paliativista*, afirmando que *la disciplina de cuidados paliativos es, en esencia, una cuestión de mirada*^1^. No puedo estar más de acuerdo con esta visión, a la que añadiría la necesidad de capacitar al resto de especialidades para que, sin necesidad de tener los mismos objetivos que el paliativista, adquieran también esa mirada, esa visión holística que permita tender puentes mucho tiempo antes, para que el paciente reciba mucho antes esos cuidados paliativos, en una continuidad asistencial que suponga que se ha dado ese diálogo del que luego, tanto pacientes como profesionales paliativistas y el propio sistema de salud, se beneficiarán. Evitar, en suma, que los cuidados paliativos lleguen tarde y supongan un inicio brusco de comunicación de malas noticias, noticias que ya eran de sobra conocidas por el equipo sanitario.

Juan Luis Torres-Tenor acuña el término *Palifobia*, refiriendo que *no es solo un problema institucional*, sino que *puede entenderse también como un mecanismo de defensa* de un *sistema sanitario entrenado únicamente para la victoria, donde quienes cuidan reciben una escasa formación en paliativos*^1^.

La formación, explicada como *capacitar a los y las profesionales sanitarios*, es una gran pieza en este puzle. La experiencia de Navarra, con la reciente colaboración de la Universidad Pública de Navarra (UPNA) con el Departamento de Salud, en el impulso de la microcredencial universitaria *Humanizar la atención a la salud*^6^, nos ha enseñado a seguir trabajando en contenidos que van desde la bioética, hasta el enfoque de género y las habilidades de comunicación. Con especial énfasis en la necesidad de planes de acogida a profesionales, donde se cuide su acompañamiento en procesos de atención a la muerte, y especial énfasis también en la necesidad de escuchar las necesidades de colectivos vulnerables, de atender a la discapacidad, a la población migrante, a las diversidades existentes con todas sus interseccionalidades (edad, sexo, etnia, etc.). Y nadie mejor que las propias personas, y las asociaciones en su derecho a la participación, para impartir esta formación.

Pero quizás el mayor impacto de esta formación ha sido el módulo de *Ética y fundamentos bioéticos de la humanización*, lo que nos hace asumir la *transversalidad* de esta materia con cualquier otra que se imparta: entender a cada ser humano de forma diferente; cada persona piensa, siente, y necesita un proyecto de vida, o una razón para vivir, cada cual la suya. Hay que explorar ese deseo de vivir en cada persona, diferenciarlo de las otras, aunque tengan la misma edad, el mismo sexo, la misma condición económica. Un deseo de vivir conformado por varias dimensiones, destacando la dimensión moral-espiritual, y la dimensión social, en su necesidad de interrelación, de vincularse, de sentirse escuchado, querido y entendido. Expuesto al dolor y al sufrimiento como característica única. Y eso nos interpela, queramos o no. Y todo ello en una sociedad que va modificando valores, que da lugar a generaciones diferentes, en muy poco espacio de tiempo. Aspectos reflejados en un libro que tuve por fin la oportunidad de leer, *La sociedad del cansancio*^7^, lectura muy recomendable.

Explorar las diferencias individuales, y también explorar las estructurales. Según los informes del Observatorio de muerte digna de Navarra^8^, la ciudadanía navarra manifiesta su deseo de fallecer en domicilio. Sin embargo, la realidad es que los fallecimientos son en mayor número en centros hospitalarios. También con la prestación de ayuda para morir se está dando este cambio: mientras que los dos primeros años se mantuvo el deseo de realizar la prestación en domicilio (trece fallecimientos en domicilio frente a uno en hospital), vemos cómo la curva va cambiando, con seis fallecimientos en el domicilio frente a cuatro en hospital durante el año 2023^9^.

Esto nos lleva a varias hipótesis. La primera, que la respuesta a las encuestas se produce -en teoría- ante una situación de buena salud de la persona encuestada; pero es la enfermedad, cuando llega, la que causa el miedo y el deseo de acudir al hospital. La segunda, que los y las profesionales que atienden en domicilio carecen de suficiente formación en cuidados paliativos, y derivan a pacientes al entorno hospitalario ante situaciones de final de vida. La tercera, que el sistema ofrece los medios, recursos y servicios necesarios para atender el final de vida en domicilio, pero aún son insuficientes. Y una cuarta, que las nuevas generaciones de personas cuidadoras, de familias y entorno, no aceptan culturalmente el fallecimiento en domicilio como antes. Podríamos elegir una hipótesis, o todas. Pero una de las herramientas de las que disponemos para revertir esta tendencia es la planificación anticipada de la atención, tal y como señalaron en esta misma revista Lina Nitola-Mendoza y Carlos Centeno en 2021^10^ y Francisco Javier Alonso y Leire González en 2018^11^, junto con el documento de voluntades anticipadas^5^. Ambos deben ir alineados y, en este sentido, todavía queda mucho recorrido, tal y como señalan Elena Antoñanzas y Lázaro Elizalde en 2022^12^.

Para ello es imprescindible entrenarnos en la deliberación. Añadir a la entrevista clínica aspectos biográficos, historias de vida. El mundo humano es un mundo de valores, señala Diego Gracia: *el ser humano es un animal deliberante, la deliberación es un proceso que le viene exigido por su propia naturaleza* (...) *que precisa de un análisis cuidadoso, con conocimiento adecuado y la adquisición de ciertas habilidades y rasgos de carácter* (...). *El buen deliberador no nace, se hace*^13^.

En conclusión, me quedo con una cita de Hannah Arendt, *La esencia humana* (…) *nace cuando la vida parte, no dejando tras de sí más que una historia*^14^. Exploremos esa historia, en aras, como dice Juan Luis Torres-Tenor, de una mirada holística que asegure unos cuidados paliativos de calidad y una buena muerte.
